# Leveraging free-text clinical records for heart disease classification through structured feature mapping

**DOI:** 10.1371/journal.pone.0354916

**Published:** 2026-07-27

**Authors:** Noha Alnazzawi

**Affiliations:** Department of Computer Science and Engineering, Yanbu Industrial College, Royal Commission for Jubail and Yanbu, Yanbu, Saudi Arabia; Polytechnic University of Marche: Universita Politecnica delle Marche, ITALY

## Abstract

Hypertension and diabetes are major risk factors for heart disease, which remains among the leading causes of morbidity and mortality worldwide. Heart disease includes heart failure, myocardial infarction, stroke, and atherosclerosis. The identification and monitoring of these risk factors are crucial for early intervention and effective management. Machine learning techniques have the potential to improve the management and prevention of heart disease by enabling the automatic detection of risk factors, which in turn can help doctors personalize treatment and facilitate preventive interventions. In this study, heart disease risk factors were automatically extracted using a combination of multimodal data: both unstructured clinical narratives (e.g., the PrevComp corpus) and structured datasets (e.g., the UCI heart disease dataset) were used to predict the presence or absence of heart disease. The classification model is based on the Light Gradient Boosting Machine (LightGBM), a state-of-the-art implementation of the gradient boosting framework that employs tree-based learning algorithms. The developed classification model demonstrated a robust predictive accuracy of 83% for the presence/absence of heart disease, supporting its potential to accurately identify high-risk patients and improve clinical outcomes.

## Introduction

According to the latest World Health Organization (WHO) report [1], more than 17.9 million people were estimated to have died in 2019 because of cardiovascular disease, accounting for 32% of all deaths globally. Among these deaths, 85% were due to heart attack or stroke. Owing to its diverse complexity and extremely high mortality rates, heart disease has become a formidable medical challenge [2].

The main risk factors that contribute to coronary artery disease (CAD) are sex, smoking, older age, family history, poor diet, lipid levels, lack of physical activity, hypertension, weight gain, and alcohol consumption [3]. Hypertension and diabetes are two examples of risk factors that can be inherited and increase the likelihood of developing heart disease [4]. Up to 75% of adults with diabetes also have hypertension, and patients with hypertension alone often show evidence of insulin resistance. Thus, hypertension and diabetes are common conditions that significantly overlap in terms of underlying risk factors and associated complications [5].

Hypertension increases the load on the heart, leading to heart muscle thinning over time. It also causes damage to the walls of blood vessels, which accelerates the development of atherosclerosis, the major cause of CAD. Over time, hypertension causes damage to the arteries, which in turn increases the risk of blood clots, strokes, and heart attacks. On the other hand, diabetes leads to the accumulation of glucose in the blood, which can damage blood vessels, accelerate atherosclerosis, and trigger inflammation in blood vessels, further promoting the buildup of fatty plaques that narrow and harden the arteries [6,7].

When present together, hypertension and diabetes significantly increase the risk of heart disease and can accelerate the progression of CAD. Notably, understanding the risk factors for heart disease in patients with hypertension and diabetes can not only guide preventive and risk-reduction measures but can also help manage patients with existing heart disease. The automatic detection of heart disease in patients with hypertension and diabetes will help doctors effectively screen for risk factors, identify patients at increased risk and facilitate early interventions for these diseases. Furthermore, automatic detection can aid the customization of treatment plans for individuals on the basis of their specific risk profiles [6,8].

However, information related to heart disease risk factors is often recorded in an unstructured format, namely free text, in electronic health records (EHRs), making data retrieval difficult and inefficient with traditional data processing methods. Advances in text mining (TM) techniques provide efficient means to automate the extraction and integration of vital information, including disease risk factors and complications, from EHRs, allowing for more effective handling of large volumes of unstructured clinical data.

The PrevComp corpus [9] consists of text obtained from the EHRs of patients known to have both hypertension and diabetes, including disease complications. For the PrevComp corpus, different machine learning techniques are used to automate the extraction and integration of disease complications from narrative text.

In contrast, the Cleveland Heart Disease dataset from the University of California Irvine (UCI) Machine Learning Repository contains structured data on heart disease risk factors. It is typically used to train machine learning models to predict the presence or absence of heart disease on the basis of various risk factors [10]. The UCI dataset includes various features that are relevant for diagnosing heart disease, such as age, sex, chest pain type, resting blood pressure, cholesterol level, fasting blood sugar, resting electrocardiographic results, maximum heart rate, exercise-induced angina, ST depression induced by exercise relative to rest, slope of the peak exercise ST segment, number of major vessels (0–3) colored by fluoroscopy, and thalassemia.

The aim of this research is to utilize advanced machine learning (ML) techniques to train an ML model on integrated multimodal data by combining structured data (e.g., the ICU dataset) with unstructured data (e.g., clinical notes from the PrevComp corpus) to provide a more comprehensive understanding of a patient’s condition. By combining these diverse data types, machine learning models can assist doctors in making more accurate and informed decisions for diagnosis, treatment, and personalized care.

Although several studies have identified the risk factors for heart disease, to our knowledge, our work represents the first attempt at integrating multimodal data (i.e., structured and unstructured) to classify narrative clinical records according to the presence or absence of heart disease as a complication of hypertension and diabetes. The contributions of this article are twofold:

1. This is the first study in which the PrevComp corpus is used to classify patient records on the basis of the presence or absence of heart disease in the patient.
2. Different ML models were trained on multimodal data via structured data (i.e., the UCI dataset) to extract heart disease risk factors from unstructured data (i.e., clinical notes from the PrevComp corpus) and, on the basis of risk factors, classify clinical records according to the presence or absence of heart disease in the patient.

## Related work

Text mining in health care is crucial because much of the relevant clinical information exists in EHRs. TM has shown significant promise in extracting heart disease risk factors from clinical records, offering the potential for better disease management, early detection, and personalized treatment [11]. In a prospective cohort study, Weng et al. [12] demonstrated that machine learning algorithms significantly enhanced the prediction of cardiovascular disease, highlighting their effectiveness and practical applicability in this domain. In the health care domain, machine learning is particularly valuable for predicting patient outcomes, such as hospital readmissions, when large, complex datasets are analyzed. This facilitates timely interventions and enhances care delivery. ML techniques have already been successfully applied to predict a variety of clinical events, such as cardiovascular events [13], sepsis [14], delirium [15], and hospital readmissions following lumbar laminectomy [16,17].

EHRs consist of two types of data, namely, structured and unstructured data—both of which provide complementary information. Structured data represent discrete, organized and predefined data, such as demographic data, laboratory results, medication lists, and diagnosis codes, whereas unstructured data provide narrative details, such as family history, chief complaints, signs and symptoms [18,19].

On the one hand, an abundance of research has been conducted on unimodal data using either structured or unstructured clinical data for heart disease prediction using different ensemble machine learning algorithms and deep learning, which have consistently shown high accuracy when different feature selection and balancing techniques are used [20–24].

For example, when structured EHR data are used, gradient boosting decision trees (GBDTs) have been widely recognized for their high accuracy and robustness in medical classification tasks, such as the prediction of heart disease. GBDT models such as extreme gradient boosting (XGBoost) [25], light gradient boosting machine (LightGBM) [26], and categorical boosting (CatBoost) [27] have demonstrated superior performance on various clinical datasets because of their ability to handle feature interactions, missing values, and heterogeneous data sources [28,29]. LightGBM [26] is a high-performance machine learning algorithm that is widely used for classification, regression, and ranking tasks [30]. As a member of the gradient boosting family, LightGBM is known for its computational efficiency, high accuracy, and low memory usage. The LightGBM algorithm achieves exceptional performance in a variety of applications, such as multiclass classification [31], click prediction [32], and learning to rank tasks [33]. In several studies, the LightGBM algorithm has been successfully applied in both classification and regression problems and has consistently revealed excellent detection results, highlighting its effectiveness as a predictive model [30]. In comparative evaluations, the LightGBM algorithm outperforms other advanced machine learning methods in various classification and diagnostic applications [10,26,30–35]. Its popularity in recent research is due not only to its predictive power but also to its fast training speed, efficient handling of large-scale data, and built-in optimization techniques [30].

On the other hand, NLP, deep learning, and transformer-based techniques have been applied to unstructured clinical narratives to predict hospital readmission [36] and to detect heart disease [37,38]. For example, the systems with the highest performance in predicting i2b2 heart disease risk factors have used ML classification algorithms, with—conditional random fields (CRFs) [39–41] and support vector machines among the most common [42–45]. However, supervised ML models require large, labeled datasets for training and can be difficult to obtain.

Recent advancements in deep learning architectures, such as bidirectional long short-term memory (BiLSTM) networks, and transformer-based models, such as BERT [46], have shown great promise in processing clinical texts because of their ability to capture semantic nuances in unstructured data [47–50]. These models leverage large-scale pretraining on biomedical and clinical corpora, enabling them to capture complex linguistic patterns and domain-specific knowledge. Examples of widely used pretrained models include BioBERT [51] and ClinicalBERT [52], which are fine-tuned on biomedical and clinical corpora and have shown promising results in extracting disease-related entities [53–56].

While previous studies on the detection of heart disease risk factors have largely relied on unimodal data (either structured or unstructured) [20–24], few studies have examined the integration of multimodal data from diverse data sources beyond a single data type (structured or unstructured) [18,24,57]. Multimodal fusion represents the combination of information from two or more types of data sources, such as clinical report types (e.g., ECG signals, echocardiograms and EHRs), allowing a more complete view of the patient’s health and therefore better accuracy, robustness, and interpretability than a single data source would. Therefore, the use of multimodal data improves understanding and enhances prediction and decision-making. In previous studies involving multimodal fusion for heart disease, image-level fusion [57] and clinical and imaging data were used to improve heart disease risk prediction [58].

Our study differs from previous work and adds value to current literature in that the PrevComp corpus was used for the first time. As mentioned earlier, the PrevComp corpus includes information from patients with both hypertension and diabetes who have heart disease as a complication. Furthermore, in this research, we exploited the advantages and superiority of LightGBM, among other tree-based techniques, to classify patients on the basis of risk factors (structured data), particularly the presence/absence of heart disease. Moreover, we used a large language model, i.e., ClinicalBERT, to process and convert clinical narrative text (unstructured data) obtained from the PrevComp corpus into structured feature values (e.g., age, chest pain, and cholesterol level) similar to those in the UCI dataset. These values were used as inputs to the trained UCI LightGBM model to test and evaluate its performance in classifying the presence or absence of heart disease.

## Materials and methods

### Dataset

The PrevComp corpus [9] consists of 274 EHRs, which are a subset of the i2b2/UTHealth 2014 Heart Disease Risk Factors dataset [39,59] for patients known to have both hypertension and diabetes. The original i2b2 dataset is a richly annotated clinical NLP resource developed to support the automatic identification, temporal tracking, and assertion classification of cardiovascular risk factors in longitudinal patient records. It contains 1,304 deidentified clinical documents from 300 patients, each annotated for key heart disease risk factors such as hypertension, hyperlipidemia, diabetes, obesity, smoking status, and CAD. Each annotation is made at the document level and includes not only temporal information—indicating whether the risk factor was present before, during, or after the document creation time (DCT)—but also assertion status, specifying whether the risk factor is affirmed, negated, or uncertain. This allows for detailed modeling of a patient’s evolving clinical profile, enabling systems to distinguish between existing conditions, ruled-out diagnoses, and possible risks.

The PrevComp corpus is annotated for mentions of complications related to interactions between hypertension and diabetes; these complications can be divided into macrovascular and microvascular disorders [5]. Microvascular complications include retinopathy, nephropathy, and neuropathy; macrovascular complications include CAD, myocardial infarction, congestive heart failure, and stroke [5,60].

Macrovascular complications include conditions related to heart disease risk factors. Usually, these conditions are expressed in free text form, e.g., stroke and coronary artery disease, which serve as indicators that the patient has a very high likelihood of developing heart disease. However, risk factors related to demographics and other numerical data related to serum cholesterol and fasting blood sugar levels are missing. Different modalities provide complementary information for detecting risk factors for heart disease and assisting in the determination of the presence of heart disease.

The UCI dataset [10] represents data collected from 303 patients referred for coronary angiography at the Cleveland Clinic between May 1981 and September 1984. The 13 independent features included those related to patient demographics, medical history, and clinical measurements. These clinical features originated from different medical reports, e.g., clinical reports, laboratory results, and electrocardiogram reports.

The UCI Heart Disease Dataset is a popular dataset used for predicting the presence of heart disease on the basis of various applicable medical attributes, such as the following:

- Age
- Sex
- Chest pain type
- Resting blood pressure
- Serum cholesterol level
- Fasting blood sugar level
- Resting electrocardiographic results
- Maximum heart rate achieved during exercise
- Exercise-induced angina
- Old peak (depression induced by exercise relative to rest)
- Depression in the ST segment
- Slope of the peak exercise ST segment
- Thalassemia

### UCI dataset classification

The UCI dataset was divided into portions: 60% for the training set, 20% for the validation set, and 20% for the test set. The training portion was used to train the algorithm, and the validation set was used for hyperparameter tuning. For hyperparameter tuning, we used exhaustive GridSearchCV with 3-fold stratified cross-validation, and the selection criterion was the highest mean F1 score across folds. Each candidate algorithm was trained on the training fold, hyperparameters were optimized via exhaustive GridSearchCV on the validation fold, and the test fold that was kept entirely separate served as the final, unbiased benchmark. All the ensemble models—LightGBM, XGBoost, CatBoost, and random forest—were wrapped in a GridSearchCV pipeline with threefold cross-validation. The hyperparameter grids (ranging from 24 to 288 combinations per model) were explicitly enumerated to ensure exhaustive and systematic exploration.

No Bayesian or randomized search strategies were used, as the hyperparameter spaces were modest in size and each evaluation was completed quickly, rendering an exhaustive search both feasible and optimal for reproducibility.

Under the same setting, we compared the performance of the proposed algorithm, LightGBM, with that of various similar tree-based ML techniques, namely, XGBoost, CatBoost and random forest. All the experiments were performed via Python and Google Colab with 25 GB of RAM available. Overall, LightGBM, which we named the UCI-trained LightGBM model, achieved the best results, as explained in the results section.

### Classification of PrevComp clinical records

The PrevComp corpus represented unstructured data that we needed to convert to a compatible format to be processed by the UCI-trained LightGBM model. Each clinical note was tokenized and passed through ClinicalBERT, and we were able to extract the 768-dimensional (CLS) token embedding from the final hidden layer as a fixed-length representation of the note. Because no ground-truth structured labels were available, we used a large language model (i.e., Meta-Llama-3.1) [61] to produce weakly supervised labels for each note in the UCI Heart Disease dataset format (age, sex, and cp). To generate weak supervision labels for the UCI feature set, Meta-Llama-3.1 was prompted using structured templates that included the complete set of 13 target patient features, their expected data types, and valid value ranges, and the model was explicitly instructed to infer each feature value from the clinical text or return null if insufficient evidence was present. The prompts constrained the response to a strict JSON format to ensure schema-compliant output, and the temperature was set to 0.1 to minimize stochastic variability. The generated responses underwent automated structural validation against the expected schema, and any outputs that failed to parse, contained missing required fields, or yielded out-of-range values were excluded during preprocessing; valid outputs were further spot-checked manually to confirm plausibility.

These LLM-generated labels were then used to train a translation network that predicts structured features directly from ClinicalBERT embeddings.

This approach acts as a knowledge distillation pipeline, where the translation model learns to infer structured features directly from embeddings. Once trained, this model allows us to map *any new note* to structured features deterministically, without invoking the LLM. This approach bridges the gap between unstructured textual data and structured machine learning pipelines, enabling the integration of rich clinical narratives into predictive models.

**Fig 1:**
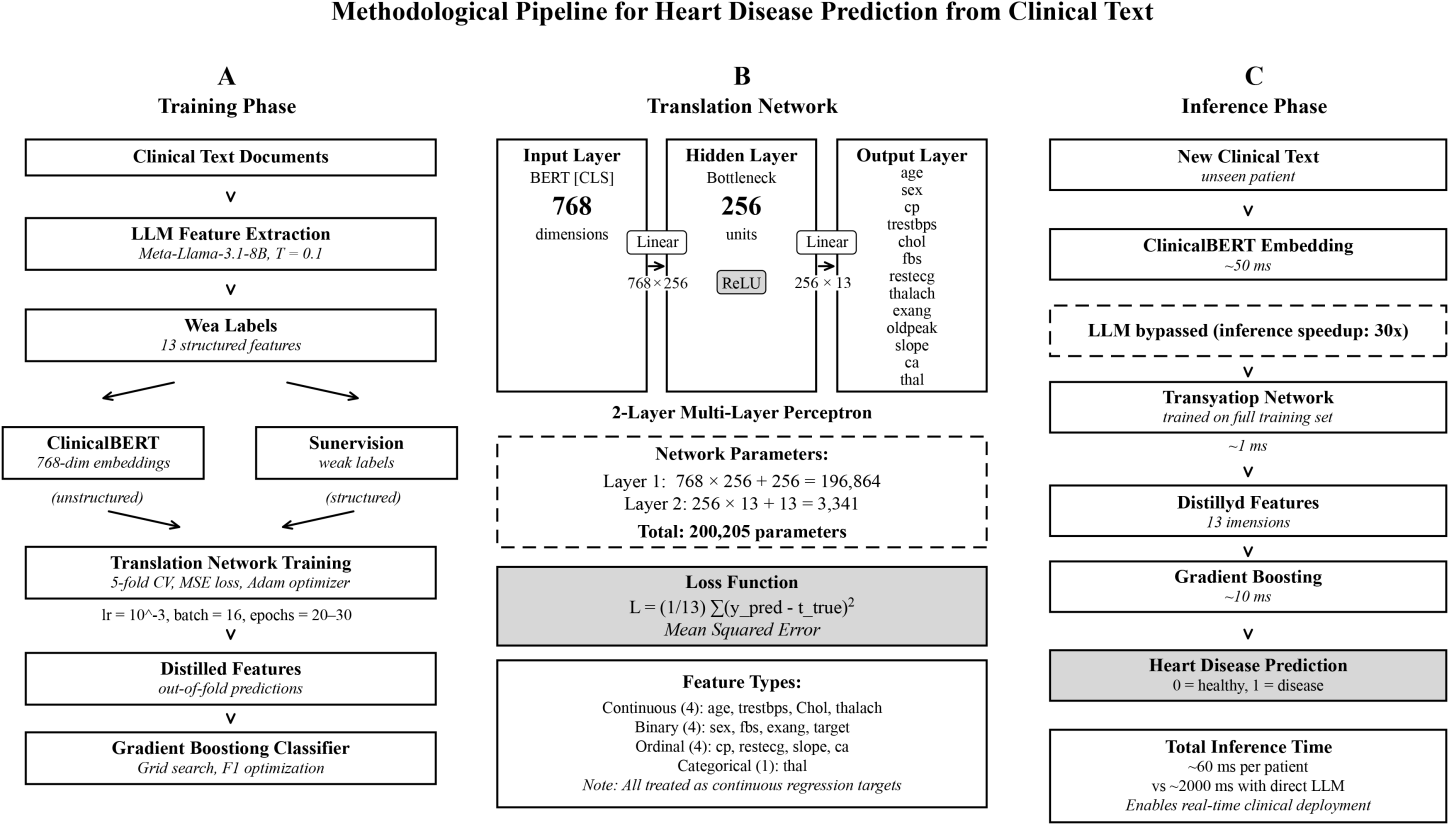
Methodological pipeline for heart disease prediction from clinical text. (A) Training phase, where the model is trained using structured clinical features and weakly supervised labels. (B) Translation network that maps structured feature representations to clinical text representations. (C) Inference phase, where the trained model predicts the presence or absence of heart disease from clinical narratives.

The extracted features from the clinical notes are then fed into the UCI-trained LightGBM model to predict the patient’s heart disease status. The UCI-trained model is capable of recognizing heart disease risk factors in clinical records from the PrevComp dataset, enabling them to classify patient records on the basis of the presence or absence of heart disease. The steps that were followed in our proposed study are shown in Fig 1 below.

## Results and discussion

The classification results of the ML models on the UCI dataset were analyzed quantitatively via performance measures, namely, precision, recall, F1 score, and accuracy [62].

Accuracy is the basic indicator for model evaluation and describes the number of correct predictions over the total number of predictions.


$$\begin{array}{c} Accuracy=number\;of\;correct\;predictions/total\;number\;of\;predictions. \end{array}$$

Precision (P) is calculated as the ratio of true positives (TPs) to the sum of true positives and false positives (FPs). Precision represents the proportion of correct positive predictions among all positive predictions made by the model.


$$\begin{array}{c} P=TP/TP+FP. \end{array}$$

Recall (R) is the percentage of true positives that are successfully identified by the model and is calculated as the ratio of TPs to the sum of TPs and false-negatives (FNs).


$$\begin{array}{c} R=TP/TP+FN. \end{array}$$

The F1 score describes the harmonic mean of both precision and recall and is calculated as follows:


$$\begin{array}{c} F1=2\times P\times R/P+R. \end{array}$$

The performance of the proposed UCI-trained LightGBM algorithm was compared with that of various similar tree-based ML techniques, namely, random forest, XGBoost, and CatBoost, as shown in Table 1.

**Table 1:** Performance of Different Machine Learning Models on UCI Datasets.

Model and class	Accuracy	Precision	Recall	F1 score
LightGBM				
0	0.83	0.86	0.73	0.79
1	0.83	0.81	0.90	0.85
Random forest				
0	0.82	0.84	0.72	0.78
1	0.82	0.80	0.89	0.84
XGBoost				
0	0.80	0.86	0.67	0.75
1	0.80	0.78	0.91	0.84
CatBoost				
0	0.80	0.81	0.72	0.76
1	0.80	0.79	0.86	0.83

The UCI-trained LightGBM model demonstrated excellent performance across various performance evaluation metrics, namely, accuracy, precision, recall, and F1 score, achieving superior performance in terms of a predictive accuracy of 83% and an F1 score of 0.82. The superiority of the UCI-trained LightGBM model can be attributed to its faster prediction speed and higher accuracy, confirming that its leafwise tree growth and native handling of missing values yielded the most robust decision boundaries for this thirteen-feature, mixed-type problem. These findings suggest that the UCI-trained LightGBM model offers increased accuracy and reliability in heart disease prediction, making it a strong candidate for addressing related clinical challenges.

To evaluate the ability of the UCI-trained model to classify the PrevComp clinical notes according to the presence or absence of heart disease, we first needed to convert the unstructured clinical notes to a structured format similar to the UCI structured dataset. Therefore, we needed to leverage contextual embeddings tailored to the health care domain. ClinicalBERT is a domain-specific variant of BERT that is pretrained on clinical text. The PrevComp clinical notes were first converted to structured feature values via ClinicalBERT, and the extracted values were mapped to match UCI heart disease dataset fields (e.g., age, chol, and cp). Afterward, the UCI-trained LightGBM model was applied to the output produced by ClinicalBERT and the final result of ClinicalBERT for the presence and absence of heart disease. The accuracy of the model’s classification was measured via accuracy metrics, and the results are shown in Table 2. According to the LightGBM model, among 274 records of the PrevComp corpus, 189 records were retained as present cases of heart disease, and 85 were absent cases of heart disease.

**Table 2:** UCI-Trained LightGBM Prediction and Doctor Verification of Cases.

Class	# UCI-trained LightGBM prediction	# Doctor verified as true
Absent	85	62
Present	189	212

agreement rate (accuracy) = (# of “correct” responses)/total # of evaluated samples

accuracy = 220/274 = 0.80

The final result of the model was verified by a cardiologist. The task of the model was explained to the doctor, and some examples were given to ensure that the doctor fully understood the task. The doctor was asked to verify the results of the model by checking the correctness of the prediction and to specify whether they agreed or disagreed. The time specified for this task was 45 days, and the doctor delivered the results on time. For further evaluation and verification, we computed the confusion matrix, as shown in Table 3. Since the PrevComp corpus is a subset of the i2b2/UTHealth 2014 shared task track 2 dataset [39], it was initially annotated for heart disease risk factors at the document level, such as CAD. The UCI-trained LightGBM model of the PrevComp corpus for the presence/absence of heart disease was compared on the basis of the initial annotations for the clinical records (i.e., 2b2/UTHealth 2014). The original annotation served as the gold standard to verify and evaluate the performance of the UCI-trained LightGBM model on the PrevComp corpus. Furthermore, the results were independently reviewed and verified by a **cardiologist** to ensure clinical accuracy.

**Table 3:** Confusion Matrix for the UCI-Trained LightGBM Model.

Actual value	Predicted value	
	**Present**	**Absent**
Present	175	37
Absent	17	45

- As shown in Table 4, when the model predicted the presence of heart disease, it was correct 91% of the time, and the model detected approximately 83% of actual heart disease cases. This means that the model correctly classified approximately **80% of all patients** (both the presence and absence of heart disease).
- Given the class imbalance in the dataset (189 vs. 85 cases), accuracy alone may provide an overly optimistic estimate of model performance. Therefore, we report the following:
- As shown in Table 5, **balanced accuracy**, which is defined as the average sensitivity and specificity, assigns equal importance to both classes and provides a more reliable evaluation when class distributions are uneven.

**Table 4:** Performance evaluation of the proposed model using precision, recall, and F1 score metrics.

Precision	Recall	F score
91.15%	82.55%	86.63%

**Table 5:** Balanced accuracy of the proposed model for heart disease prediction.

Specificity	Sensitivity	Balanced accuracy
72.58%	82.55%	77.57%

Regardless of the strong performance of the UCI-trained LightGBM model, in some cases, the model failed to classify clinical records correctly and produced false classification for either the presence or absence of heart disease for a given patient record. The main reason for the false-negatives of the model was ambiguous language in expressing specific risk factors and symptoms in the clinical record, whereas the ML model relied on the explicit expression of symptoms, e.g., severe chest pain, and failed to recognize risk factors that required indirect inference to be recognized, e.g., nonspecific chest discomfort. Therefore, in such cases, the clinical records were incorrectly misclassified as “absent” or negative for heart disease.

In another example, a record included the information that the “EKG showed nonspecific ST-T changes,” which was misclassified as absent. When verifying the classification of this record, the doctor clarified that the patient’s record should be classified as “present” or a positive heart disease case instead of “absent.” This occurred because the doctor read the patient’s clinical records and recognized that even though EKG findings of the patient did not reveal ST-T changes, the patient presented shortness of breath—defined as “tired more easily when climbing up the stairs or walking 2 blocks than previously.” In addition, diabetes, unexplained nausea, sweating and old age together suggested that the patient had CAD. Another example was the clinical record stating that “The patient reports occasional chest tightness, which seems related to anxiety. There was no clear evidence of ischemic changes on the ECG.” This record was misclassified as a positive case because the information is strongly related and associated with heart disease; however, the risk factor for chest tightness was not related to heart disease but rather was attributed to a psychological condition, i.e., anxiety. In most cases, for false positives, the model confused overlapping markers such as “chest pain” and “shortness of breath”, which match the signs and symptoms of heart disease, and the records were misclassified as indicating heart disease in such cases.

Though encouraging results were obtained, there are a number of limitations that should be taken into account when interpreting the findings. Firstly, the PrevComp corpus consists of only 274 clinical records, meaning that the statistical power and any predictions based on past observations of the suggested approach across wider patient populations and healthcare institutions could well be limited. Secondly, the UCI Heart Disease dataset, while extensively used as a benchmark for the prediction of cardiovascular disease, was created in the period between 1981 and 1984. As a results, the dataset might not fully represent current clinical practice, patient demographics, advances in diagnostic technologies, treatment strategies, or the standards for today’s EHR documentation. Consequently, these factors may affect the external validity of models trained on such data when applied to modern healthcare environments these factors may have an adverse effect on the external validity of models trained on such data when applied to current healthcare environments. As a result, the results should be interpreted as evidence of the feasibility of the proposed knowledge-transfer framework rather than definitive proof of how well the results of a clinical study or experiment can be applied to a larger population, or different settings. Future studies should assess the approach through the adoption of current larger and more diverse multi-institutional EHR datasets to better establish its strength and applicability in real-world clinical settings.

## Conclusions

The coexistence of hypertension and diabetes requires an integrated, multidisciplinary approach to reduce the risk of heart disease and other complications.

In this study, we developed a classification model based on the gradient boosting machine (LightGBM) algorithm to automatically extract and recognize heart disease risk factors from patients’ health records. The model demonstrated a robust predictive accuracy of 83% for the presence/absence of heart disease compared with that of other tree-based learning algorithms when the UCI heart disease dataset was applied. Our proposed model was further tested to classify clinical records from the PrevComp corpus to determine the presence/absence of heart disease and achieved promising results, with an accuracy of 0.80. Our proposed model has the potential to assist health care providers in early detection, personalized treatment, and effective management of heart risk. Early detection, effective treatment and lifestyle interventions play vital roles in reducing the risk and improving the prognosis for these patients, helping doctors provide better care and ultimately enhancing patient outcomes.

However, challenges related to data privacy remain and must be addressed to fully realize the potential of text mining in clinical practice. Continued research on improving the accuracy, scalability, and transparency of these text mining techniques will be crucial for enhancing patient care and reducing the burden of heart disease. In addition to increasing the risk of heart disease, the co-occurrence of hypertension and diabetes can contribute to a range of serious complications, such as kidney damage, retinopathy, neuropathy, and a heightened risk of stroke.

One of the key limitations of the proposed model is its cross-disease generalization, especially because the model is trained on clinical data collected between 1981 and 1984. As a result, diagnostic criteria, treatment protocols, and patient demographics may differ substantially from those observed in contemporary clinical practice. Additionally, compared with modern EHRs, the dataset contains a relatively limited set of structured variables.

Furthermore, models trained on specific datasets may struggle to perform well across different health care settings, thereby limiting their broader applicability. Addressing this issue is critical for ensuring robust and reliable clinical decision support. As such, an important direction for future research is to focus on enhancing the model’s ability to generalize across a wide range of diseases and datasets, with the goal of improving its overall predictive accuracy, clinical utility, and reliability in supporting diagnosis and prevention in various medical contexts.
